# Tracing Responses to a Syndemic: A Systematic Map of Substance Use, Mental and Sexual Health Interventions for Gay, Bisexual and Other Men Who Have Sex With Men in Southeast Asia

**DOI:** 10.1002/jia2.70198

**Published:** 2026-11-14

**Authors:** T. Charles Witzel, Disayanun Pejpongsai, Worawalan Waratworawan, Nattharat Samoh, Nattapat Jatupornpimol, Adam Bourne, Lu Gram, Nittaya Phanuphak, Gloria Lai, Siripong Srichau, Harry Prabowo, Alison J. Rodger, Thomas E. Guadamuz

**Affiliations:** ^1^ Mahidol Center for Health, Behavior and Society Mahidol University Bangkok Thailand; ^2^ Institute for Global Health University College London London UK; ^3^ Institute of HIV Research and Innovation Bangkok Thailand; ^4^ Australian Research Centre in Sex Health and Society La Trobe University Melbourne Victoria Australia; ^5^ International Drug Policy Consortium Bangkok Thailand; ^6^ Service Workers In Group Foundation Bangkok Thailand; ^7^ Asia Pacific Network of People Living with HIV/AIDS Bangkok Thailand

**Keywords:** bisexual and other men who have sex with men, gay, mental health, sexual health, Southeast Asia, substance use, syndemics

## Abstract

**Introduction:**

In Southeast Asia, a triple syndemic of substance use, poor mental and sexual health is shaped by regional political, economic and sociocultural environments and disproportionately affects gay, bisexual and other men who have sex with men (GBMSM). Yet, most GBMSM implementation research, regionally and globally, focuses narrowly on HIV prevention and care, with little attention to mental health and substance use.

This review aimed to systematically map interventions addressing these interlinked areas of GBMSM wellbeing in Southeast Asia, identify evidence gaps and inform the design of holistic, syndemic‐responsive interventions.

**Methods:**

Following PRISMA‐ScR guidelines, we searched six databases (01/2010−06/2024) for intervention/evaluation studies including GBMSM (cis and trans) in Southeast Asia, reporting outcomes on substance use, mental and/or sexual health. We completed citation/full text screening and data extraction in duplicate. We mapped interventions by type and availability of outcome data.

**Results:**

We identified 2016 unique records; 142 publications reported 130 studies describing 148 interventions reaching 154,502 GBMSM.

Interventions focused primarily on sexual health (*n* = 143), concentrated along the care continuum. This included facility‐based HIV testing (*n* = 25), community‐based HIV testing (*n* = 14), HIV self‐testing (*n* = 13), assisted partner notification (*n* = 1), pre‐exposure prophylaxis (*n* = 29), post‐exposure prophylaxis (*n* = 3), HIV treatment (*n* = 20), sexually transmitted infection/hepatitis C virus testing/treatment (*n* = 12) and vaccination (*n* = 2). Fewer interventions were behavioural and psychosocial, including psychosocial support (*n* = 7), education (*n* = 11) and outreach (*n* = 3). Three took miscellaneous approaches.

Few interventions primarily focused on mental health and substance use. Three addressed mental health: two using psychosocial strategies, one of which had a dual sexual health focus, and one assessing depression following HIV treatment initiation. Two addressed substance use, both with psychosocial approaches. Mental health outcome data (primary/secondary) were presented in nine interventions; substance use in five, mostly as secondary measures. Only one intervention reported outcomes spanning all three syndemic domains.

**Discussion:**

These findings reflect a narrow intervention landscape, shaped partly by funder priorities, that fails to fully address the intersecting challenges GBMSM face with substance use, mental and sexual health.

**Conclusions:**

In the rebuilding period following the closure of USAID and many US NIH‐funded research initiatives, intervention development should take a broader view, crafting holistic services ameliorating multiple challenges. These must attend to local priorities and take account of regional contexts exacerbating the syndemic.

**Abbreviations**
BBV: blood‐borne virus
GBMSM: gay, bisexual and other men who have sex with men
HBV: hepatitis B virus
HCV: hepatitis C virus
HIV: human immunodeficiency virus
LGBTQ+: lesbian, gay, bisexual, transgender, queer (and other identities)
NIH: National Institutes of Health
PEP: post‐exposure prophylaxis
PrEP: pre‐exposure prophylaxis
PRISMA: Preferred Reporting Items for Systematic Reviews and Meta‐Analyses
SPiDER: Sample, Phenomenon of interest, Design, Evaluation, Research type
STI: sexually transmitted infection
UK: United Kingdom
US: United States
USAID: United States Agency for International Development
WHO: World Health Organization

## 1 Introduction

Research among gay, bisexual and other men who have sex with men (GBMSM) and other populations facing marginalization globally is increasingly concerned with syndemics: co‐occurring and mutually reinforcing epidemics which occur in specific temporal and geographical contexts [1]. In Southeast Asia, GBMSM face a triple syndemic of substance use, poor mental health and sexual health shaped by the region's unique socioeconomic, political, historical and cultural environments [2, 3, 4]. Syndemic theory requires two or more co‐occurring epidemics that interact synergistically, with harms worsened by adverse social and structural conditions [1]. Syndemics are shaped by high‐level factors such as legal and structural oppression, financial barriers and policy exclusion, which contribute to worsening health and wellbeing of groups made vulnerable [5, 6, 7, 8, 9, 10], necessitating interventions grounded in the contexts, values and priorities of those affected.

HIV among GBMSM in Southeast Asia is a critical public health priority. A recent systematic review found a pooled HIV prevalence of 7.38% (95% CI: 4.83–9.93) among GBMSM in the region [11] compared with a general adult prevalence of approximately 0.2%–0.3% [12]; this represents a roughly 25‐ to 35‐fold disparity. Consequently, HIV prevention, treatment and sexual health, including blood‐borne viruses (BBVs) and sexually transmitted infections (STIs), have been extensively studied in this region [2, 4, 11, 13, 14, 15, 16]. Evidence of the triple syndemic is strongest in Malaysia and Thailand, where longitudinal and review evidence demonstrate syndemic associations between HIV acquisition risk and mental health challenges with links to drug and alcohol use [4, 17, 18].

Comparatively less attention has been paid to mental health and substance use, both key elements of wellbeing in this population [19, 20, 21, 22]. Mental health conditions among GBMSM can be reinforced by minority stressors [5, 23, 24], especially homophobia and social rejection [25]. Economic exclusion, punitive policies criminalizing sex between men and strong stigma around homosexuality can also contribute to depression and anxiety [2, 14, 26]. A recent systematic review including 25 Southeast Asian studies (40% from Thailand) among LGBTQ+ people found greater mental health challenges than among cisgender heterosexual counterparts [27], with depression prevalence ranging from 23.5% to 40.3% and lifetime suicidal ideation from 16.0% to 58.9% [27].

Substance use is common among GBMSM communities worldwide [28, 29, 30, 31], ranging from recreational drugs and alcohol consumption to contexts specific to sexual activity [32, 33, 34, 35, 36]. A recent systematic review of 49 publications exploring sexualized drug use among GBMSM in East and South Asia included only 14 studies from Southeast Asia [37]. These reported a pooled 12‐month prevalence of sexualized drug engagement of 13%–15%, though definitions of sexualized drug use varied considerably across studies, adding uncertainty to prevalence estimates around consumption of specific drugs [37]. Notably, some substances (e.g. methamphetamine and other stimulants) are associated with depression, anxiety and psychosis [38, 39, 40], while mental health challenges can drive substance use as a coping mechanism—a bidirectional relationship underpinning the syndemic [41]. Finally, drug use is highly criminalized across Southeast Asia with severe punitive measures including imprisonment and forced‐rehabilitation programmes [36, 42, 43]. Criminalization harms wellbeing directly and by constraining what research and service provision is possible, and whether individuals feel able to access support or take part in research.

GBMSM in Southeast Asia, therefore, face potentially heightened impacts from the triple syndemic [5, 26, 44], underscoring the critical need for interventions addressing multiple support areas. Interventions addressing any single domain remain valuable because the causal pathways are likely interlinked, but it is also vital to understand how interventions might work across the triple syndemic by addressing multiple priority areas simultaneously.

Despite Southeast Asia having a long history of innovative HIV implementation research, with a substantial proportion historically funded by US overseas development aid, it is unclear to what degree other elements of this syndemic have been measured and addressed in research and service provision. This is especially important given the contemporary shift to developing and delivering multipronged responses to the HIV epidemic [45]. Interventions addressing the HIV prevention and care cascade have become increasingly complex, with many comprising multiple components supporting wellbeing beyond sexual health [46]. This is exemplified by approaches integrating mental health support into HIV programming [47, 48]. It is unclear, however, to what extent this approach has extended to Southeast Asia, where priorities may be constrained by legislative environments and shaped by donor agencies rather than by the communities themselves.

This study aims to systematically map interventions implemented to address the constituent elements of the triple syndemic of substance use, mental health and sexual health among GBMSM (cis and trans) in Southeast Asia.

The specific objectives are to:

- map what types of interventions have been implemented and in which countries
- understand to what degree outcomes relating to substance use, mental health and sexual health have been included in implementation research
- identify critical priorities for future implementation research addressing the triple syndemic

## 2 Methods

We used the PRISMA Scoping Review extension guidance, acknowledging that systematic mapping and scoping reviews share nearly identical search and screening methodologies [49, 50]. Systematic mapping is a process of collecting, collating and describing the parameters of the existing literature on a particular topic [51], and is especially useful for identifying both paucity of research and potential saturation. Our protocol is available online [52]. This systematic map draws exclusively on previously published data; therefore, ethical approval was not required, nor was informed consent.

### 2.1 Search Strategy

The search was conducted based on a Sample, Phenomenon of interest, Design, Evaluation, Research type (SPiDER) framework developed for this study (Supplementary 1). The review was conducted between March 2024 and November 2025. Searches were conducted from 1/01/2010 to 26/06/2024 on six databases (PUBMED, SCOPUS, MEDLINE [EBSCO], EMBASE, PsycINFO and Web of Science), focusing on evidence from the onset of the biomedical HIV prevention era, marked by the first publication on the efficacy of HIV pre‐exposure prophylaxis (PrEP), as well as increasing focus on GBMSM mental health in Southeast Asia [27, 53]. Search terms focused on population (GBMSM in Southeast Asia), the phenomenon of interest (substance use, mental and sexual health), study design (interventions, routine programming) and evaluation/research type (study design, outcomes of interest). See Table S1 for SPiDER framework and protocol for full search terms [52].

### 2.2 Inclusion/Exclusion Criteria

Eligible publications include those which report outcomes (qualitative or quantitative) in one or more of the priority areas (substance use/mental/sexual health) from intervention implementation (e.g. pilots, demonstration projects, routine service provision, trials, evaluation) for GBMSM. We defined GBMSM as individuals who identify as men and who have sex with other (self‐identified) men. Studies from Southeast Asian countries (Brunei, Cambodia, Indonesia, Laos, Malaysia, Myanmar, the Philippines, Singapore, Timor‐Leste, Thailand and Vietnam) were eligible.

#### 2.2.1 Inclusion Criteria

- Original studies published in peer‐reviewed journals and conference abstracts which have been published and indexed
- Empirical studies published in English starting from 1/01/2010 to 26/06/2024;
- Reporting data on intervention implementation and/or outcomes related to substance use, sexual and mental health
- Focusing on GBMSM (cis/trans) in Southeast Asia

#### 2.2.2 Exclusion Criteria

- Published before 1/01/2010
- Published in languages other than English
- Research from outside Southeast Asia
- Non‐GBMSM populations
- Review, theoretical studies and other studies with no empirical data, and no outcome data

Studies which included GBMSM alongside other populations were eligible if they provided disaggregated data for GBMSM in Southeast Asia, or if the GBMSM population constituted >90% of all included participants. This threshold was chosen to increase confidence that outcomes mapped are representative of GBMSM rather than other communities with distinct needs (e.g. transgender women).

### 2.3 Screening Process

We utilized double‐screening, with each article undergoing independent review by two authors following inclusion/exclusion criteria. During the title and abstract stage, studies were categorized as follows:

- Not relevant (clearly does not meet inclusion criteria)
- Maybe relevant (possibly meets inclusion criteria)
- Eligible (appears to meet inclusion criteria)

Disagreements were resolved through team discussion (TCW, DP, NS, WW). Studies classified as maybe relevant and eligible proceeded to full‐text screening, which was conducted in duplicate using a screening tool (see protocol [52]). Ineligible studies were classified by reason for ineligibility.

### 2.4 Data Extraction

Data were extracted by one team member (DP, NS or WW) and checked by the first author (TCW). Areas extracted include citation details (title, year of publication), study details (country, intervention, aim, primary focus, secondary focus, study design, setting, participants) and intervention domains (intervention description, control description [where relevant]), theoretical grounding, inclusion/exclusion, method of recruitment, number of participants, outcome types, mental health outcomes, substance use outcomes, sexual health outcomes.

Interventions were determined as having a *primary focus* on a particular area of the syndemic (substance use, mental and/or sexual health) if they included a focus on outcomes related to a relevant domain in the primary aim, or if the design of the intervention was to elicit or understand changes in this area. We defined substance use as the consumption of psychoactive substances (alcohol or other drugs but not nicotine) and mental health as relating to common or severe mental disorders, as well as psychological wellbeing. Interventions were deemed to have *any focus or data collection* if their publications specifically described a focus on the area or if relevant data were collected, irrespective of whether changes were measured and/or described.

We systematically extracted the following outcome data for further analytic classification (see below). *Intervention delivery outcomes*: intervention acceptability (all data related to intervention acceptability from participants), barriers and facilitators, uptake (how many participants who were offered an intervention accessed it). *Outcome data*: any measurement of changes related to substance use, mental or sexual health (including knowledge and behaviour) because of the intervention. Sexual health outcomes were further broken down into knowledge and behaviour change (e.g. increases in knowledge, change in behaviour), testing outcomes (any data related to test results, test algorithm performance), prevention outcomes (e.g. HIV/STI incidence, linkage to prevention, PrEP outcomes, post‐exposure prophylaxis [PEP] outcomes), treatment interventions (e.g. linkage to care, retention in care, viral suppression).

### 2.5 Analysis

Consistent with systematic mapping approaches, we did not conduct a quality assessment [51, 54, 55]. All data were exported from Covidence into Microsoft Excel.

Following extraction, interventions were classified by the first author into categories based on the general intervention approach described using the extracted data. For complex interventions which included multiple components, the main aim of the research report was used for this categorization. For example, interventions which combined HIV testing, PrEP and psychosocial components (e.g. adherence counselling) were categorized as PrEP interventions if the primary aim of the paper was to explore PrEP outcomes. For papers reporting interventions addressing the entire HIV prevention and care cascade, including HIV testing, prevention and/or antiretroviral therapy, these were categorized based on the first step on the HIV care continuum reported, usually HIV testing.

Where results from single studies/interventions were reported across multiple articles and/or conference abstracts, we used the publications which reported the most comprehensive account of study outcomes as the primary study publication for mapping purposes. All publications reporting multiple interventions had data from each intervention disaggregated.

We classified extracted data related to sexual health, mental health and substance use as outcome data if there was data about change (positive, negative or none) related to the outcome domain (substance use, mental or sexual health) following intervention exposure or experience.

Intervention focus was tabulated overall, by primary focus, any focus or data collection and by outcome data using Microsoft Excel. This was input into tables by geography, by intervention type, and by intervention type and outcome availability, using EndNote for the latter.

## 3 Results

Our searches yielded 5834 citations, of which 3818 were duplicate records, which we removed. We screened the remaining 2016 records by title and abstract; 355 proceeded to full‐text review, where an additional 213 were excluded. In total, 142 publications were included in the systematic map; because eight studies were reported across multiple publications (*n* = 12), these 142 publications corresponded to 130 unique studies, describing 148 interventions including a total of 154,502 GBMSM (cis and trans) participants. See Figure 1 for the PRISMA flow chart.

**FIGURE 1:**
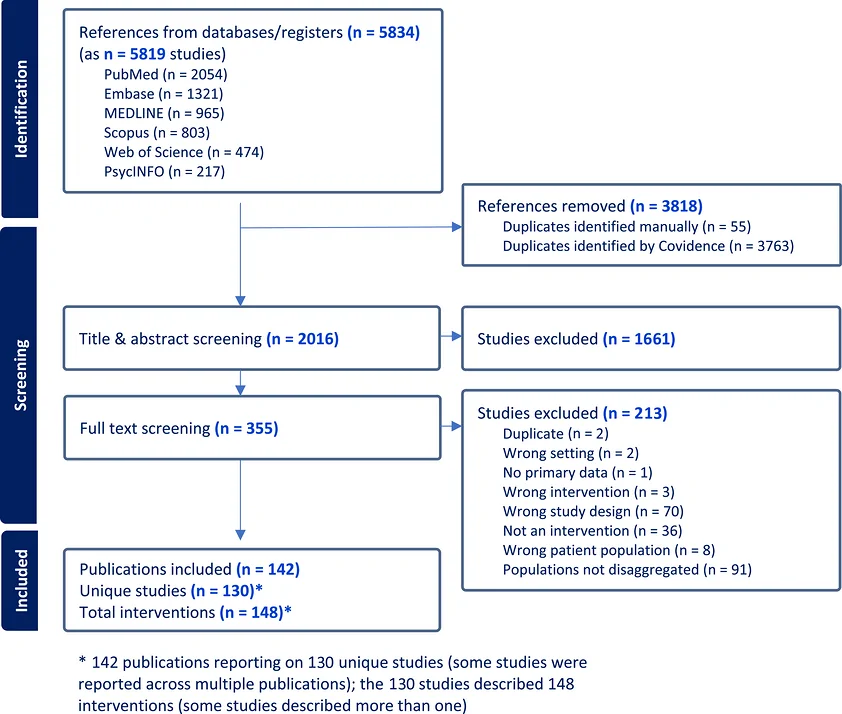
PRISMA flowchart.

### 3.1 Intervention Focus and Outcome Data

Almost all interventions (*n* = 143, 96.6%) focused primarily on sexual health, and one had a dual primary focus on mental and sexual health. A further two had a primary focus on mental health (total *n* = 3, 2.0%), and two on substance use (1.4%).

While a further 21 interventions collected data or described a focus on mental health (total *n* = 24), secondary outcome data were only presented for six of these, meaning primary or secondary outcome data were presented for nine interventions in total. In terms of substance use, although 41 interventions collected data or described a focus on this area, secondary outcome data were only available from three additional interventions, for a total of five reporting any primary or secondary outcome data. Only one intervention (primarily focused on sexual health) reported outcome data from all three syndemic areas. Interventions reporting mental health and substance use outcomes were more recent; all emerged from 2014 and most (8/12) from 2020 onwards.

The majority of interventions overall (*n* = 78, 52.7%) were from Thailand, with fewer from Vietnam (*n* = 17, 11.5%), Indonesia (*n* = 15, 10.1%), Philippines (*n* = 12, 8.1%), Malaysia (*n* = 11, 7.4%), Singapore (*n* = 8, 5.4%), Myanmar (*n* = 4, 2.7%) and Cambodia (*n* = 3, 2.0%). No studies were found from Brunei Darussalam, Lao PDR or Timor‐Leste. See Table 1 and Figures S1–S3.

**TABLE 1:** Interventions by availability of primary or secondary outcome data by geography.

Country	Sexual health	Mental health	Substance use	Total
Brunei Darussalam	**—**	**—**	**—**	**—**
Cambodia	3	**—**	**—**	3
Indonesia	15	1	**—**	15
Lao PDR	**—**	**—**	**—**	**—**
Malaysia	10	3	1	11
Myanmar	4	**—**	**—**	4
Philippines	12	**—**	**—**	12
Singapore	8	1	1	8
Timor‐Leste	**—**	**—**	**—**	**—**
Thailand	78	4	3	78
Vietnam	17	**—**	**—**	17

For studies reporting mental health outcome data, the same pattern persisted. The highest concentration of interventions was from Thailand (*n* = 4, 44.4%), followed by Malaysia (*n* = 3, 33.3%), with Indonesia and Singapore each having one (11.1%).

Studies reporting substance use outcomes were rarer, with more than half of the research in Thailand (*n* = 3, 60.0%), with one study from each of Malaysia and Singapore (20.0%).

### 3.2 Intervention Types by Focus Area

Of 148 interventions found in total, 106 focused on the HIV prevention and care cascade, 14 focused on other BBVs and STIs, 25 were behavioural and psychosocial and three were other types of interventions. Below, we describe intervention types by syndemic area and primary focus. Table 2 outlines all intervention types by syndemic area.

**TABLE 2:** Intervention types by primary focus.

		All	Sexual health	Mental health	Substance use
Intervention areas	Intervention types	(*n* = 148)	(*n* = 143)	(*n* = 3)	(*n* = 2)
**HIV prevention and care**	**HIV testing in facilities**	**(*n* = 25)**	**(*n* = 25)**	**(*n* = 0)**	**(*n* = 0)**
**cascade (*n* = 106)**	**HIV testing in facilities (general)**	**22**	**22**	**—**	**—**
	**HIV testing in facilities (peer‐led/delivered)**	**3**	**3**	**—**	**—**
	**HIV testing in communities**	**(*n* = 14)**	**(*n* = 14)**	**(*n* = 0)**	**(*n* = 0)**
	**HIV testing in communities (general)**	**11**	**11**	**—**	**—**
	**HIV testing in communities (peer‐led/delivered)**	**3**	**3**	**—**	**—**
	**HIV self‐testing**	**(*n* = 13)**	**(*n* = 13)**	**(*n* = 0)**	**(*n* = 0)**
	**HIV self‐testing (facility‐based)**	**2**	**2**	**—**	**—**
	**HIV self‐testing (online/mail)**	**1**	**1**	**—**	**—**
	**HIV self‐testing (peer‐led/delivered)**	**7**	**7**	**—**	**—**
	**HIV self‐testing (other)**	**3**	**3**	**—**	**—**
	**Assisted partner notification**	**(*n* = 1)**	**(*n* = 1)**	**(*n* = 0)**	**(*n* = 0)**
	**PrEP**	**(*n* = 29)**	**(*n* = 29)**	**(*n* = 0)**	**(*n* = 0)**
	**PrEP (general)**	**23**	**23**	**—**	**—**
	**PrEP (peer‐led/delivered)**	**6**	**6**	**—**	**—**
	**PEP**	**(*n* = 3)**	**(*n* = 3)**	**(*n* = 0)**	**(*n* = 0)**
	**PEP (general)**	**3**	**3**		
	**HIV treatment**	**(*n* = 21)**	**(*n* = 20)**	**(*n* = 1)**	**(*n* = 0)**
	**HIV treatment (general facility‐based)**	**20**	**19**	**1**	**—**
	**HIV treatment (peer delivered)**	**1**	**1**	**—**	**—**
**BBVs and STIs (*n* = 14)**	**HCV testing**	**(*n* = 3)**	**(*n* = 3)**	**(*n* = 0)**	**(*n* = 0)**
	**HCV testing in communities**	**1**	**1**	**—**	**—**
	**HCV self‐testing**	**2**	**2**	**—**	**—**
	**STI testing and treatment**	**(*n* = 9)**	**(*n* = 9)**	**(*n* = 0)**	**(*n* = 0)**
	**STI testing**	**8**	**8**	**—**	**—**
	**STI treatment**	**1**	**1**	**—**	**—**
	**BBV and STI vaccination**	**(*n* = 2)**	**(*n* = 2)**	**(*n* = 0)**	**(*n* = 0)**
	**HBV vaccination**	**2**	**2**		
**Behavioural and**	**Education**	**(*n* = 11)**	**(*n* = 11)**	**(*n* = 0)**	**(*n* = 0)**
**psychosocial (*n* = 25)**	**Education**	**1**	**1**	**—**	**—**
	**Online education**	**8**	**8**	**—**	**—**
	**Peer education**	**2**	**2**	**—**	**—**
	**Psychosocial support**	**(*n* = 11)**	**(*n* = 8)**	**(*n* = 2)**	**(*n* = 2)**
	**Counselling**	**3**	**1**	**1**	**1**
	**Group support**	**3**	**3** ^a^	**1** ^a^	**—**
	**Online psychosocial support**	**4**	**4**	**—**	**—**
	**Peer‐led/delivered psychosocial support**	**1**	**0**	**—**	**1**
	**Outreach**	**(*n* = 3)**	**(*n* = 3)**	**(*n* = 0)**	**(*n* = 0)**
	**Outreach (peer‐led/delivered**)	**3**	**3**	**—**	**—**
**Others (*n* = 3)**	**Others** ^b^	**(*n* = 3)**	**(*n* = 3)**	**(*n* = 0)**	**(*n* = 0)**

^a^ One intervention had a dual focus on sexual and mental health.

^b^ Provision of supplies and materials, health service redesign and a human rights‐focused intervention.

#### 3.2.1 Sexual Health

The vast majority of interventions focused primarily on sexual health. Of all sexual health‐focused interventions, most (*n* = 105) clustered on the HIV prevention and care cascade. These included HIV testing in facilities (*n* = 25), in communities (*n* = 14), self‐testing (*n* = 13), assisted partner notification (*n* = 1), PrEP (*n* = 29), PEP (*n* = 3) and HIV treatment (*n* = 20). In general, intervention designs were highly heterogeneous, with differing approaches used depending on context and communities served. Although many HIV prevention and care cascade interventions were facility‐based, these also expanded care into the public sphere through community‐based testing and HIV self‐testing. Of the HIV testing and care cascade interventions, 20 were peer‐led/delivered.

Fewer interventions (*n* = 14) focused on STIs and other BBVs; these included STI testing and treatment (*n* = 9), hepatitis B vaccination (*n* = 2) and hepatitis C virus (HCV) testing (*n* = 3). STI testing and treatment tended to take place in facilities; however, all three HCV testing interventions took place in communities or through self‐testing. It should be noted that many HIV prevention and care interventions incorporated BBV and STI testing as part of complex intervention approaches.

In total, we found 21 sexual health‐focused interventions which were primarily behavioural and/or psychosocial: 11 educational, seven psychosocial and three outreach. The majority of sexual health‐focused education interventions were online (*n* = 8), as were four of the psychosocial and one of the outreach interventions. Psychosocial interventions used a range of approaches including group support (*n* = 2) and counselling (*n* = 1). Similar to BBV and STI interventions, many HIV prevention and care cascade interventions utilized psychosocial and/or behavioural components as part of complex approaches.

Finally, there was one sexual health‐focused intervention each using other approaches: provision of supplies and materials, health service redesign and a human‐rights‐focused intervention. No HCV treatment interventions were identified.

A further three interventions included secondary sexual health outcome data. These included one HIV treatment intervention and two psychosocial (counselling) interventions.

#### 3.2.2 Mental Health

We found three interventions which had a primary mental health focus (one of which had a dual primary focus on sexual health). One of these interventions (published across two papers) examined HIV treatment; one was a group support intervention and one a counselling intervention.

A further six interventions reported secondary mental health outcomes. These included one each of the following interventions: online psychosocial support, online education, group support, peer support, counselling and HIV treatment.

#### 3.2.3 Substance Use

Two interventions (both psychosocial) primarily focused on substance use. One provided counselling and the other peer support. A further three interventions reported substance use secondary outcome data. These included one educational intervention, one counselling and one online psychosocial support intervention.

### 3.3 Intervention Outcome Mapping

Below, we describe mapping results by intervention type (see Table 3).

**TABLE 3:** Intervention mapping by outcomes (primary and secondary).

	Intervention delivery			Sexual health outcomes				Mental health	Substance use
	Acceptability	Barriers and facilitators	Uptake	Knowledge and behaviour change	Testing	Prevention	Treatment	Outcomes	Outcomes
**Clinical interventions**									
HIV testing in facilities (incl. peer‐led/delivered)	*n* = 5 [56, 57, 58, 59, 60]	*n* = 10 [56, 57, 58, 59, 60, 61, 62, 63, 64]	*n* = 13 [57, 59, 62, 63, 65, 66, 67, 68, 69, 70, 71, 72, 73]	*n* = 4 [58, 59, 71, 74]	*n* = 18 [56, 62, 65, 66, 67, 69, 70, 71, 73, 74, 75, 76, 77, 78, 79, 80, 81]	*n* = 2 [74, 77]	*n* = 4 [74, 79, 80]		
HIV testing in communities (incl. peer‐led/delivered)	*n* = 3 [82, 83, 84]	*n* = 1 [82]	*n* = 4 [69, 82, 85, 86]		*n* = 12 [69, 82, 83, 85, 87, 88, 89, 90, 91, 92, 93]		*n* = 5 [82, 83, 84, 87, 92]		
HIV self‐testing (all approaches)	*n* = 8 [63, 66, 83, 94, 95, 96, 97]	*n* = 2 [63, 94]	*n* = 9 [63, 66, 72, 80, 92, 95, 98, 99, 100, 101]		*n* = 12 [66, 80, 83, 92, 95, 96, 97, 98, 99, 100, 101]	*n* = 1 [97]	*n* = 9 [66, 80, 83, 92, 96, 97, 98, 100]		
Assisted partner notification					*n* = 1 [92]		*n* = 1 [92]		
PrEP (all approaches)	*n* = 5 [102, 103, 104, 105, 106, 107]	*n* = 13 [103, 104, 105, 106, 107, 108, 109, 110, 111, 112, 113]	*n* = 15 [102, 104, 107, 109, 111, 112, 113, 114, 115, 116, 117, 118, 119, 120, 121]		*n* = 5 [111, 114, 118, 122, 123]	*n* = 17 [102, 109, 111, 113, 114, 115, 118, 119, 121, 122, 123, 124, 125, 126, 127, 128, 129, 130, 131]	*n* = 1 [123]		
PEP	*n* = 2 [132, 133]	*n* = 2 [132, 133]		*n* = 1 [133]		*n* = 2 [133, 134]			
HIV treatment (incl. peer‐led/delivered)	*n* = 2 [135, 136]	*n* = 4 [135, 136, 137, 138]	*n* = 6 [139, 140, 141, 142, 143]			*n* = 1 [144]	*n* = 16 [137, 140, 141, 142, 143, 145, 146, 147, 148, 149, 150, 151, 152, 153]	*n* = 2 [137, 145, 154]	
HCV testing (incl. self‐testing)	*n* = 2 [155, 156]				*n* = 2 [87, 155]		*n* = 1 [87]		
STI testing and treatment					*n* = 8 [157, 158, 159, 160, 161, 162]		*n* = 2 [162, 163]		
Vaccination			*n* = 2 [77, 164]			*n* = 2 [77, 164]			
**Behavioural and psychosocial**									
Education (all types)	*n* = 7 [165, 166, 167, 168, 169, 170, 171]		*n* = 6 [165, 166, 167, 168, 171, 172]	*n* = 7 [165, 166, 170, 171, 173, 174, 175]	*n* = 5 [165, 166, 171, 172, 174]	*n* = 1 [170]		*n* = 1 [165]	*n* = 1 [173]
Outreach		*n* = 1 [176]	*n* = 1 [177]	*n* = 2 [176, 178]	*n* = 2 [177, 178]		*n* = 1 [177]		
Psychosocial support (all types)	*n* = 6 [179, 180, 181, 182, 183, 184]	*n* = 3 [179, 180, 185]	*n* = 2 [185, 186]	*n* = 5 [180, 182, 183, 184, 186]	*n* = 2 [181, 183]	*n* = 5 [181, 182, 183, 186, 187]	*n* = 5 [180, 183, 184, 186, 188]	*n* = 6 [179, 180, 181, 186, 188, 189]	*n* = 4 [182, 185, 186, 189]
**Others**									
Others^a^	*n* = 1 [190]	*n* = 1 [190]	*n* = 2 [190, 191]	*n* = 1 [192]	*n* = 1 [191]				

^a^ Provision of supplies and materials, health service redesign and a human rights focused intervention.

#### 3.3.1 HIV Prevention and Care Cascade

Intervention and sexual health outcome data on the HIV prevention and care cascade were very well developed. All intervention types had evidence around acceptability as well as barriers and facilitators. Except for PEP interventions, all had data related to uptake. In terms of sexual health outcomes, knowledge and behaviour change was less comprehensively measured, with only HIV testing in facilities and PEP interventions reporting in these areas. In contrast, HIV testing outcomes were present for most interventions, as were HIV treatment outcomes. Prevention outcomes were more rarely described in HIV testing (including HIV self‐testing) and treatment interventions, but were very well developed for PrEP and PEP.

Only two studies reported outcome data related to mental health as part of HIV prevention and care, and none on substance use. These included a study on mental health among GBMSM diagnosed with HIV receiving clinical care [137], and one study exploring the impact of antiretroviral therapy on the mental health of GBMSM (two publications) [145, 154].

#### 3.3.2 Other BBVs and STIs

Fewer interventions (*n* = 14) presented data on various aspects of HCV/HBV and STIs. In this category, acceptability outcomes were only present for HCV testing interventions, with no studies reporting on intervention delivery barriers and facilitators and only two on uptake, both of which described HBV vaccination interventions. Consistent with reported aims, in this category, studies describing testing interventions tended to report treatment outcomes and vaccination interventions prevention outcomes.

No studies reported on mental health or substance use outcomes following interventions primarily addressing other BBVs and STIs.

#### 3.3.3 Behavioural and Psychosocial Interventions

The 25 behavioural and psychosocial interventions reported a very wide range of outcomes. Education and psychosocial support had a well‐developed evidence base around acceptability and uptake. Barriers and facilitators were reported for outreach, and for psychosocial support. Knowledge and behaviour, testing, prevention and treatment outcomes were present across all three intervention sub‐types, except for prevention outcomes from outreach interventions.

One study reported on an educational intervention and mental health outcomes, specifically on self‐esteem following engagement with a sexual health‐focused mobile educational app in Indonesia [165]. An online education intervention reported substance use outcomes [173].

One study on a psychosocial intervention reported data in all three syndemic areas, and a further one on both mental health and substance use [186, 189]. Four interventions using psychosocial approaches reported mental health and two substance use outcomes.

The intervention reporting on all three syndemic areas used motivational interviewing to support GBMSM. This primarily addressed sexual risk and alcohol use but also reported mental health outcomes [186]. A peer‐led/delivered intervention aimed to reduce chemsex engagement and reported both substance use and mental health outcomes [189].

Psychosocial interventions reporting mental health outcomes included a study reporting on counselling for GBMSM living with HIV in Malaysia which was described as a requirement of HIV treatment [179], a study on a self‐management programme for GBMSM living with HIV in Thailand [180], a study on group cognitive behavioural therapy for GBMSM living with HIV in Thailand [188] and an e‐health psychosocial support‐focused sexual health app in Malaysia [181].

Psychosocial interventions reporting substance use outcomes included an evaluation of a chemsex support service in Singapore [185], and a randomized trial reporting outcomes from an online educational video and e‐counselling intervention for GBMSM not living with HIV in Thailand [182].

## 4 Discussion

Our systematic map identified 148 interventions from 130 studies, which included 154,502 GBMSM and reported outcomes related to sexual health, mental health and substance use. Almost all had a primary focus on HIV and sexual health, and very few reported mental health and substance use outcomes. Interventions were highly diverse; approaches varied by implementation context and communities served. More than half were from Thailand, with fewer from Vietnam, Indonesia, Philippines, Malaysia, Myanmar, Singapore and Cambodia. No eligible studies were found from Brunei Darussalam, Lao PDR or Timor‐Leste. Over two‐thirds (*n* = 106, 71.6%) were on the HIV prevention and care cascade, with fewer addressing other BBVs and STIs or using psychosocial/behavioural approaches.

Although nearly a third of interventions (*n* = 46, 31.1%) included a stated focus or collected data on more than one syndemic area, few presented outcome data related to mental health or substance use. Where this was presented, it was sometimes not disaggregated by population. Among interventions that collected mental health data, just over a third (9/24, 37.5%) presented outcomes usable for this review. The proportion presenting substance use outcomes was much lower (5/41, 12.2%) because very few interventions sought to address substance use, and in those that measured this domain, data were presented at baseline only, included as an explanatory variable in effectiveness of sexual health interventions or not disaggregated by population. This indicates that although these areas are measured, actual changes following intervention engagement are not reported routinely, or not disaggregated by population when they are. Studies that did report such data were more likely to be published recently, indicating that practice may be changing. Indeed, the first paper found reporting substance use outcomes was from 2014 [173]; with mental health outcomes published the following year [186]. Nevertheless, in a syndemic‐responsive landscape, these data would be expected to be published, particularly for complex interventions with multiple psychosocial components.

Consistent with the volume of interventions, outcomes data related to the HIV prevention and care cascade were well developed; however, less than 5% of these interventions presented disaggregated secondary outcome data related to mental health and none substance use. There were fewer interventions primarily focused on addressing BBV and STIs; however, many HIV prevention and care cascade interventions included BBV and STI testing and treatment. Several psychosocial/behavioural interventions reported mental health and substance use outcomes. However, this evidence base remains very limited.

The strong focus on developing the evidence base for sexual health interventions and lack of attention to mental health and substance use is likely reinforced by structural and cultural factors. First, and most importantly, the long‐established political economy of development—whereby donor agencies set priorities carried through from planning to funding allocation and implementation research—profoundly shapes research and service provision priorities [40, 193, 194]. The historical primacy of the United States in supporting health interventions in many Southeast Asian countries means that much of what service providers would like to address, especially around substance use, is not possible because of donor restrictions, and when it is possible, it is not prioritized [40]. Further, intense criminalization of drugs in Southeast Asia makes substance use research and service provision legally challenging, especially in countries such as Malaysia and Singapore [185, 195]. Finally, there is intense stigma surrounding mental health challenges in Southeast Asia [196, 197]; this may make researchers reluctant to ask questions about psychological wellbeing, and communities reticent to answer them.

Although intensely painful for service providers and affected communities, the closure of USAID presents a mid‐term opportunity to reshape priorities in service provision; stabilization and rebuilding efforts in countries most affected can aim to more holistically address the needs of communities in ways not previously possible [198, 199].

There are substantial evidence gaps which must be addressed. First, and most obviously, there is a worrying paucity of interventions aiming to support mental health and substance use. This is critical given the complex relationship between substance use and mental health, itself a key syndemic driver [41, 200, 201, 202]. Very few interventions (*n* = 12, 8.1%) presented primary or secondary outcome data related to these domains. This is surprising given these areas were routinely measured in many studies. Given their independent morbidity and links to HIV acquisition, building this evidence base is a priority. A recent study surveying 43 sexual and gender minority focused community‐based organizations in the Philippines, Indonesia, Singapore and Thailand found that less than half had any focus on mental health issues, with the most common service provided being screening and referral, with funding acting as a major barrier [203]. Therefore, both mental health and substance use interventions must be developed, and this must be coupled with substantial funding for implementation. While these critical gaps remain, it is questionable whether ending the HIV pandemic can be successful [4, 17, 204, 205].

Second, interventions reporting substance use or mental health outcome data were typically siloed by serostatus: those describing mental health outcomes primarily included GBMSM living with HIV, while interventions reporting substance use outcomes primarily included GBMSM without an HIV diagnosis. Addressing both substance use and mental health together has further benefits around addressing highly interconnected, intersectional issues in a holistic way, potentially improving outcomes [206]. This means substance use and mental health interventions must become less serostatus‐specific and more person‐centred, thereby meeting a broader range of needs.

Third, the greatest concentration of research among GBMSM in Southeast Asia is from Thailand, reflecting the country's position as a productive site of public health research [207]. While other countries, especially Vietnam, Malaysia, Singapore and the Philippines, have substantial research originating from within the country, Brunei Darussalam, Cambodia, Laos, Myanmar and Timor‐Leste are under‐represented. Capacity for implementation research should be developed within these countries as economic, political and cultural contexts impact delivery and effectiveness.

Fourth, there are several sexual health interventions which appear under‐researched. These include HCV testing/treatment, critical in the drive to eliminate HCV [208]. No evidence was found on mpox vaccination implementation, and none on doxyPEP/PrEP [209, 210]. Finally, given HIV self‐testing's potential utility in expanding PrEP service delivery, developing the evidence around linkage to PrEP following self‐testing is a priority [211, 212].

Fifth, although peer‐led/delivered approaches are becoming more common, further developing and refining these remains an under‐explored opportunity. Peer‐led/delivered services may have key benefits in terms of reducing stigma, decreasing medicalization, increasing intervention acceptability, while also potentially reducing costs, all of which will be important in developing mental health and substance use responsive interventions [213]. Because Southeast Asia has a leadership role in this area, there is huge opportunity to expand peer‐led/delivered approaches to focus on the entire syndemic, holistically improving health and wellbeing while empowering communities and reducing HIV incidence.

Finally, although GBMSM in Southeast Asia who use drugs face intense harms related to criminalization [36, 40, 214], no interventions on addressing the legal impacts of substance use were found despite this type of support being valued by affected communities [215]. Developing and robustly evaluating such interventions is a priority, and a further area where Southeast Asia can lead.

This is, to our knowledge, the first attempt to map intervention approaches addressing the triple syndemic for GBMSM in Southeast Asia. This will provide a vital roadmap for researchers, practitioners and policymakers to develop genuinely syndemic responsive interventions, a much‐needed innovation in the region. We note some limitations.

To focus on the unique needs of GBMSM in Southeast Asia, we excluded studies which included GBMSM (cis and trans) alongside transgender women unless data were disaggregated by population or if GBMSM represented 90% or greater of the study participants. This led to a large volume of otherwise‐eligible publications (*n* = 91) being excluded, reflecting historical trends in HIV research whereby transgender women were often subsumed under the label GBMSM [216]. While this approach has slowly been changing in response to community pressure, oftentimes data from the two groups are still presented as one, potentially obscuring the needs and intervention outcomes of these groups.

We used systematic mapping to explore where outcome data exist across a very wide range of interventions. Because of the volume of research and enormous heterogeneity of outcome measures found in this evidence base, it is not possible to present outcome data.

Because of resource constraints, we only included English language publications in academic sources. This means that potentially relevant examples in other languages and/or published as grey literature were not captured, potentially introducing systematic bias. In particular, we may have missed relevant interventions from community organizations published in languages other than English.

Finally, in the region, sexualized drug use (particularly chemsex/*hi‐fun*) has been the focus of much recent public health concern. Potentially promising interventions from just outside the region (Hong Kong and Taiwan) have been published recently [217, 218]. Further, the Asia‐Pacific Chemsex Symposium provides a key catalyst for innovation in this area [219], and more implementation research is likely to be formally published soon. As this emerges, systematic reviewing should consolidate and explore this evidence base.

## 5 Conclusions

Our systematic map found very few interventions addressing mental health and substance use for GBMSM in Southeast Asia. Our findings reflect a narrow intervention landscape—shaped by funder priorities and legislative contexts—that fails to fully address the intersecting challenges GBMSM face with substance use, mental and sexual health, constraining holistic responses and limiting choice. In the stabilization and rebuilding period following the closure of USAID, it is imperative that intervention development for GBMSM in Southeast Asia takes a broader view, crafting holistic services responding to the triple syndemic. A first step could be the incorporation of psychosocial components spanning all three syndemic domains in complex interventions regardless of serostatus, thereby addressing the siloing of mental health and substance use. Community members should be meaningfully involved in the designing and delivery of interventions, ensuring new support strategies attend to local priorities and take account of the regional contexts exacerbating the syndemic.

New funding models are required. South−South initiatives could make use of resource pooling, especially via multilateral organizations active in the region. Members of affected communities must be embedded within any new funding structures and fully empowered with decision‐making capacity within them.

## Author Contributions

*Study conceptualization*: TCW, AJR and TEG. *Funding*: TCW. *Protocol development*: TCW, DP and WW. *Searches and screening*: TCW, DP, WW and NS. *Data extraction*: TCW, DP, WW, NS and NJ. *Data analysis*: TCW. *Data interpretation*: TCW, DP, WW, NS, NJ, AB, LG, NP, GL, SS, HP, AJR and TEG. *Drafting*: TCW and AJR. *Critical input*: TCW, DP, WW, NS, NJ, AB, LG, NP, GL, SS, HP, AJR and TEG.

## Funding

This work was supported by the Wellcome Trust [226345/Z/22/Z]. LG was supported by the Wellcome Trust [331938/Z/25/Z]. TEG was supported by the U.S. National Institute of Mental Health [R01MH119015 and R34MH123337].

## Conflicts of Interest

All authors declare no conflicts of interest.

## Supporting information




**Supporting File 1**: jia270198‐sup‐0001‐SuppMat.docx.

## Data Availability

The data that support the findings of this study are openly available on the study website following publication at https://www.tm.mahidol.ac.th/muhbs/systematic‐map/.
