# Supplementary material for: Tracing Responses to a Syndemic: A Systematic Map of Substance Use, Mental and Sexual Health Interventions for Gay, Bisexual and Other Men Who Have Sex With Men in Southeast Asia
Source: J Int AIDS Soc. 2026 Nov 14;29(11):e70198. doi: 10.1002/jia2.70198 (PMC13768991; doi:10.1002/jia2.70198)
Supplement: Supplementary file 1 [file JIA2-29-e70198-s001.docx]

Supplementary material

Supplementary table 1. SPiDER framework underpinning search strategy

| Area | Inclusion criteria |
| --- | --- |
| **Sample** | Gay, bisexual and other men who have sex with men (cis and trans) in Association of Southeast Asian countries |
| **Phenomenon of interest** | Syndemics (substance use, mental and sexual health) |
| **Design** | Studies reporting intervention implementation or evaluation |
| **Evaluation** | Reports intervention outcome data related to substance use, mental and/or sexual health |
| **Research type** | Observational (qualitative, quantitative and mixed-methods studies), interventional (randomised control trial or quasi-randomised control trial) |

Supplementary figure 1: All interventions by country

Supplementary figure 2: Interventions reporting mental health outcome data (primary or secondary) by country

Supplementary figure 3: Interventions reporting substance use outcome data (primary or secondary) by country
